# IFN-γ amplifies NFκB-dependent *Neisseria meningitidis* invasion of epithelial cells via specific upregulation of CEA-related cell adhesion molecule 1

**DOI:** 10.1111/j.1462-5822.2007.01038.x

**Published:** 2007-08-30

**Authors:** Natalie J Griffiths, Christopher J Bradley, Robert S Heyderman, Mumtaz Virji

**Affiliations:** 1 Department of Cellular and Molecular Medicine, School of Medical Sciences, University of BristolBristol, BS8 1TD, UK; 2 ReNeuron Ltd, 10 Nugent Road, Surrey Research ParkGuildford, Surrey, GU2 7AF, UK; 3 Malawi-Liverpool-Wellcome Trust Clinical Research ProgrammePO Box 30096, Chichiri, Blantyre 3, Malawi

## Abstract

Temporal relationship between viral and bacterial infections has been observed, and may arise via the action of virus-induced inflammatory cytokines. These, by upregulating epithelial receptors targeted by bacteria, may encourage greater bacterial infiltration. In this study, human epithelial cells exposed to interferon-gamma but not tumour necrosis factor-alpha or interleukin 1-beta supported increased meningococcal adhesion and invasion. The increase was related to Opa but not Opc or pili adhesin expression. *De novo* synthesis of carcinoembryonic antigen-related cell adhesion molecule 1 (CEACAM1), a major Opa receptor, occurred in epithelial cells exposed to the cytokine, or when infected with Opa-expressing bacteria. Cell line-dependent differences in invasion that were observed could be correlated with CEACAM expression levels. There was also evidence for Opa/pili synergism leading to high levels of monolayer infiltration by capsulate bacteria. The use of nuclear factor-kappa B (NFκB) inhibitors, diferuloylmethane (curcumin) and SN50, abrogated bacterial infiltration of both untreated and interferon-gamma-treated cells. The studies demonstrate the importance of CEACAMs as mediators of increased cellular invasion under conditions of inflammation and bring to light the potential role of NFκB pathway in Opa-mediated invasion by meningococci. The data imply that cell-surface remodelling by virally induced cytokines could be one factor that increases host susceptibility to bacterial infection.

## Introduction

Several epidemiological studies have reported spatial and temporal association between specific bacterial and viral infections of the human upper respiratory tract (Hament *et al*., 1999; Beadling and Slifka, 2004). Susceptibility to infection by frequent colonizers, including *Neisseria meningitidis, Haemophilus influenzae* and *Moraxella catarrhalis*, increases markedly following influenza and/or respiratory syncytial virus (RSV) infections (Cartwright *et al*., 1991; Cartwright, 1995; Hament *et al*., 1999; Beadling and Slifka, 2004). The insight into the underlying mechanisms that alter the disposition of these commensals is far from complete. One mechanism may involve upregulation of specific host receptors targeted by bacteria. Many receptors are normally expressed at low levels and may be upregulated in response to inflammatory cytokines (Dansky-Ullmann *et al*., 1995; Fahlgren *et al*., 2003). Such upregulation, by increasing the affinity of bacteria–host interactions, may encourage bacterial infiltration (Tran Van Nhieu and Isberg, 1993; Bradley *et al*., 2005; Rowe *et al*., 2007).

In addition to permissive host conditions, bacterial attributes that may modulate their virulence potential include adhesins and surface polysaccharides. In the case of *N. meningitides*, pili and Opa proteins appear to determine host and tissue specificity via targeting human-specific molecules (Virji *et al*., 1992; 1996a; Merz and So, 2000). Pili, by virtue of their morphology, traverse the capsule and are effective primary adhesins in all meningococcal serogroups (Stephens and McGee, 1981; Virji *et al*., 1991; Nassif and So, 1995; Merz and So, 2000). Meningococcal pili may target human CD46, but this needs further confirmation (Kallstrom *et al*., 1997; Tobiason and Seifert, 2001; Gill *et al*., 2003; Edwards and Apicella, 2005; Kirchner and Meyer, 2005; Kirchner *et al*., 2005). CD46, a complement regulatory protein, is expressed on most cells and may be upregulated by certain cytokines (Bjorge *et al*., 1996). Such upregulation on human target cells for meningococci and the consequences on bacterial pilus-mediated interactions are not known.

Meningococcal outer membrane proteins Opa and Opc become effective adhesins only in acapsulate bacteria. The Opc protein targets integrins via their ligands such as vitronectin and fibronectin (Virji *et al*., 1994; Unkmeir *et al*., 2002). In addition, both Opa and Opc may bind heparan sulfate proteoglycans (HSPGs) (de Vries *et al*., 1996; 1998; Virji *et al*., 1999).

The Opa proteins belong to an antigenically and phase-variable family of adhesins encoded by three to four independent genes in *N. meningitidis* and by up to 11 genes in *N. gonorrhoeae* (Aho *et al*., 1991; Bhat *et al*., 1991; Hobbs *et al*., 1994). Despite the antigenic variation, distinct Opa proteins can bind to a domain common to several human carcinoembryonic antigen-related cell adhesion molecules (CEACAMs) (Virji *et al*., 1996b; 1999). *N. meningitidis* Opa–CEACAM interactions occur most effectively with acapsulate phenotypes (Virji *et al*., 1996a; 1999). This observation, and similar inhibitory effect of surface sialic acids on integral outer membrane adhesin function observed in other studies also, has lead to the postulation that downmodulation of capsule must occur during intimate adhesion and invasion of the host (Stephens *et al*., 1993; Virji *et al*., 1993a; 1995a; Hammerschmidt *et al*., 1996; Read *et al*., 1996; Virji, 1996; de Vries *et al*., 1996; Deghmane *et al*., 2000; Hardy *et al*., 2000). As acapsulate meningococci are highly susceptible to serum bactericidal activity, following blood infiltration, capsule upregulation or selection of capsulate phase variants would be required for survival. However, more recent investigations using cell lines transfected with human CEACAM1 gene demonstrated Opa-mediated adhesion of apparently fully capsulate bacteria to cells expressing high levels of CEACAM1 (Virji *et al*., 1996b; Bradley *et al*., 2005; Rowe *et al*., 2007).

In order to address how remodelling of target cell surfaces together with key bacterial attributes (pili, Opa, Opc, capsule) may affect breaching of an intact target monolayer by virulent phenotypes, in the current studies, human epithelial cells were treated with cytokines to mimic conditions that may follow virally-induced inflammation. The studies demonstrate the primary importance of Opa–CEACAM interactions in such a system and the increased capacity of capsulate bacteria to infect cytokine-stimulated target cells. This cellular invasion could be abrogated by anti-CEACAM antagonists including the CEACAM binding recombinant polypeptide (rD-7; Hill *et al*., 2005), as well as anti-inflammatory agent curcumin and SN50, a peptide that prevents nuclear factor-kappa B (NFκΒ)-mediated cellular functions.

## Results

### Increased association of *N. meningitidis* with interferon-gamma (IFN-γ)-stimulated cells is mediated primarily via the Opa proteins

**Fig. 1:**
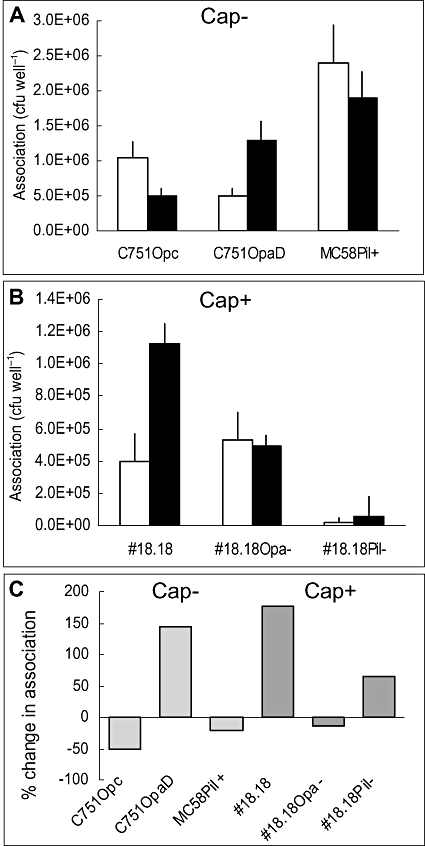
Increased association of Opa-expressing but not Opc- or pili-expressing bacteria with IFN-γ-stimulated Chang cells. Chang cells were treated with IFN-γ (filled columns) or left untreated (blank columns). The meningococcal phenotypes employed are described in Table 1. Three acapsulate (Cap^–^) Nm isolates expressing either Opc, OpaD (strain C751) or pili (strain MC58) (A), or capsulate (Cap^+^) derivatives of MC58#18.18 with Opa and pili adhesins or those lacking Opa or pili (B), were used to infect confluent monolayers, and associated bacteria were quantified by viable count assays. All #18.18 derivatives expressed Opc and LPS of L3 immunotype.A and B. Means and SEs of representatives of several experiments are shown.C. Mean values of per cent change in association of the acapsulate and capsulate variants after IFN-γ treatment compiled from data of 3–4 independent experiments are shown; SEs were within 25% of the means. Per cent change: (test − control/control) × 100.

Chang conjunctiva epithelial cells were chosen as the first model system in which to address the above questions, as the cell line is known to have the capacity to express receptors for the meningococcal pili, Opa and Opc adhesins (Virji *et al*., 1992; 1993a;). Moreover, as IFN-γ, a key inflammatory cytokine, has been shown to increase adhesion receptor expression in a variety of cell lines (Kantor *et al*., 1989; Fahlgren *et al*., 2003), we first examined whether IFN-γ increased expression of receptors for any of the three major meningococcal ligands on Chang cell surface. For this purpose, Chang cells were pretreated with IFN-γ for 24 h, and the binding of a number of defined capsulate and acapsulate Nm phenotypes was studied (Table 1, Fig. 1). The data showed a consistent increase in binding of acapsulate Opa-expressing bacteria to IFN-γ-stimulated cells, but such effect was not observed on the binding of Opc-expressing bacteria or acapsulate piliated bacteria without Opa or Opc proteins (Fig. 1A).

**Table 1:** Meningococcal derivatives and their characteristics.

Strain/derivative	Capsule	pili	Opc	Opa
C751				
C751Op^–^	–	–	–	–
C751OpaD	–	–	–	OpaD^+^
C751Opc	–	–	+	–
MC58				
MC58 Pil^+^ (¢17)	–	+	–	–
#18.18	+	+	+	OpaB^+^
#18.18Opa^–^	+	+	+	–
#18.18Pil^–^	+	–	+	OpaB^+^

In order to assess how IFN-γ treatment affected adhesion of Opa-expressing and Opa-deficient capsulate bacteria, we used several derivatives of the serogroup B strain MC58 isolate #18.18 (Pil^+^, Opa^+^, Opc^+^) as well as its Opa^–^ and pil^–^ derivatives (Table 1). In this case also, increased adhesion of Opa-expressing but not Opa-deficient bacteria was observed when using IFN-γ-treated cells. Moreover, as for acapsulate phenotype, although pili clearly contribute significantly to Chang cell adhesion, pilus-mediated adhesion of #18.18Opa^–^ (Pil^+^ Opc^+^ phenotype) did not increase after IFN-γ treatment (Fig. 1B). It is noteworthy that, in unstimulated Chang cells, adhesion appears to be primarily due to pili, whereas with IFN-γ-stimulated cells, significant increase in binding of Opa^+^ Pil^+^ bacteria (#18.18), but not Opa^–^ Pil^+^ (#18.18Opa^–^), bacteria can be seen. In the absence of pili (#18.18Pil^–^), bacterial adhesion is greatly reduced for untreated Chang cells. Further, even though the binding of this phenotype is modest, increased interactions can be seen with IFN-γ-stimulated cells (Fig. 1B and C). Comparison of the three #18.18 isolates suggest the role of pili in potentiating adhesion of capsulate phenotypes via the Opa proteins in IFN-γ-stimulated cells. This is illustrated further below.

### Contribution of CEACAM and HSPG adhesion receptors to cellular interactions of Opa-expressing bacteria

**Fig. 2:**
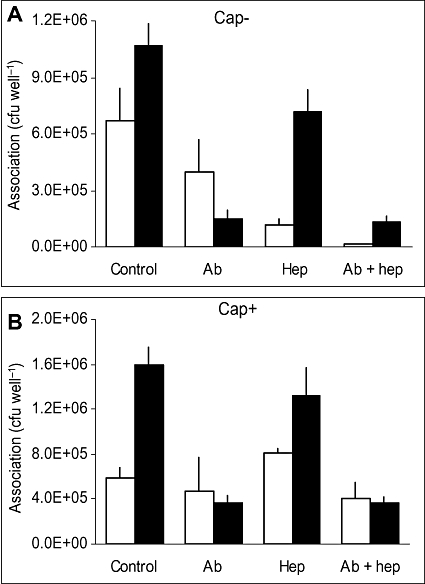
Assessment of the contribution of HSPGs and CEACAMs to increased Opa-dependent binding of IFN-γ-stimulated Chang cells. IFN-γ-treated cells (filled columns) and untreated cells (blank columns) were infected with acapsulate C751OpaD variant (A) or capsulate #18.18 (B) in the presence of 50 μg ml^−1^ heparin (hep), rabbit anti-CEACAM antibody AO115 (Ab) or a mixture of antibody and heparin (Ab + hep). AO115 reacts with CEA, CEACAM1 and CEACAM6. Normal rabbit serum used as a control in some tests showed no inhibition (not shown). Means and SEs of one representative of several experiments are shown.

Opa proteins have been shown to interact with HSPGs on resting Chang cells, and this interaction can be inhibited significantly by incorporation of heparin in the infection medium (van Putten *et al*., 1997; Virji *et al*., 1999). To assess the contribution of CEACAMs versus HSPGs in bacterial interactions with cytokine-stimulated cells, the studies incorporated heparin and anti-CEACAM antibody in the infection medium. Although heparin reduced acapsulate bacterial interactions both with stimulated and unstimulated cells, it was the CEACAM-blocking antibody that had the greater effect on IFN-γ-treated cells (Fig. 2A). With capsulate #18.18 that may adhere via pili, the inhibition of adhesion to unstimulated cells was not observed with heparin and was modest with anti-CEACAM antibody. With cytokine-treated cells, the antibody caused significant inhibition of cellular interactions (Fig. 2B). A similar inhibition was also observed using rD-7, a recombinant molecule known to block Opa-mediated binding to CEACAMs (Hill *et al*., 2005) (data not shown), suggesting the more prominent involvement of Opa–CEACAM interactions in IFN-γ-stimulated cells and potentiation of Opa function by pili.

The conclusion that pili potentiate Opa-mediated adhesion of capsulate bacteria, and not vice versa, is based on the fact that pili traverse the capsule and are functional in capsulate phenotypes, whereas Opa-expression alone is largely ineffective in mediating efficient interactions of capsulate phenotypes. That pilus function is not affected whether or not cells are stimulated is clear from Fig. 1B (#18.18 Opa^–^); only the Opa function is augmented on cell stimulation as demonstrated by the use of CEACAM antagonists. This suggests that Opa function is aided by the presence of pili.

### Carcinoembryonic antigen-related cell adhesion molecule (CEACAM) expression by cytokine-treated cells

**Fig. 3:**
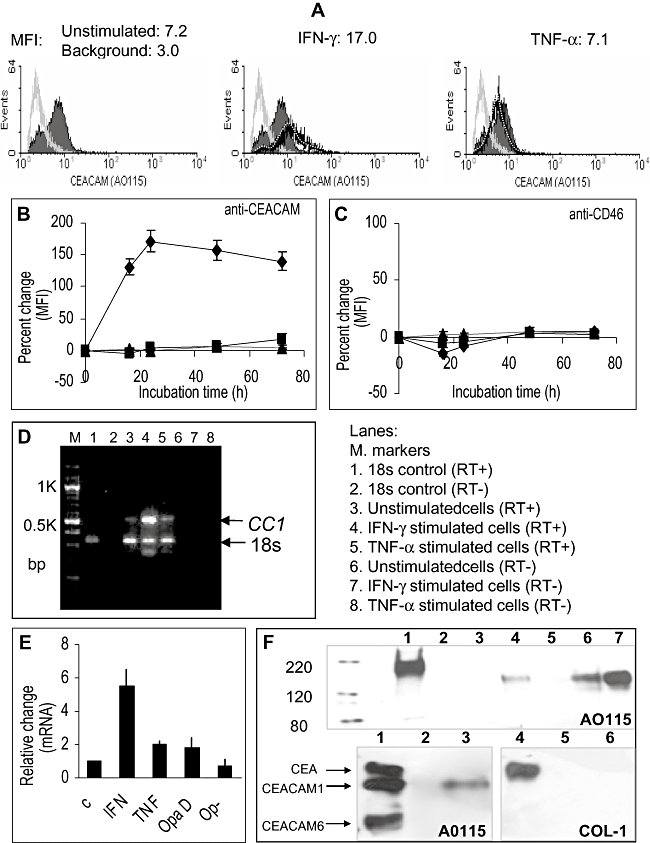
Surface expression and *de novo* transcription of CEACAMs in Chang cells following cytokine stimulation and bacterial infection. Surface expression of CEACAMs on Chang cells was assessed by flow cytometry before and after cytokine stimulation using anti-CEACAM antibody AO115 that binds to multiple CEACAMs. Examples of histograms of CEACAM expression from one experiment are shown (A). CEACAM expression of unstimulated cells is shown in black (filled profile), that of stimulated cells in dotted white lines (traced on to black unfilled profile), and binding of the secondary antibody alone is shown in grey.B and C. Per cent change in the expression of CEACAM and CD46 receptors in response to various cytokines as determined by flow cytometry. AO115 was used to detect CEACAMs (B) and J4-48 for CD46 detection (C) in Chang cells exposed to IFN-γ (diamonds), TNF-α (squares) and IL-1β (triangles) over a 72 h period. Per cent change of MFI observed over untreated cells is shown. Data are means and SEs from three determinations. Note: A and B are separate experiments.D. Agarose gel profile showing the results of a typical semiquantitative RT-PCR of mRNA extracted from Chang cells illustrating the relative levels of 18s rRNA and *ceacam1* mRNA present in unstimulated and cytokine-stimulated cells. Lane contents are shown on the right. RT, reverse transcriptase.E. The relative changes in *ceacam1* mRNA in cytokine-stimulated Chang cells (24 h) or in cells infected with bacteria (3 h) were calculated after normalizing for 18s rRNA. Means and SEs of 2–4 experiments are shown.F. Western blots showing CEACAM proteins expressed in Chang cells.Top: proteins extracted from unstimulated Chang cells [lane 2 (10 μg), lane 3 (20 μg) and lane 5 (40 μg)] or those exposed to IFN-γ[lane 4 (20 μg) and lane 6 (40 μg)] were analysed by Western blotting using anti-CEACAM antibodies. AO115 binds to multiple CEACAMs but recognized a protein only in stimulated cells, which corresponded to the migration of CEACAM1. Control samples of transfected HeLa cells expressing distinct CEACAMs were used for comparison (lane 1: HeLa-CEA and lane 7: HeLa-CEACAM1; 4 μg total protein of each).Bottom: lanes 1–3: polyclonal AO115 and anti-CEA/CEACAM3 cross-reacting monoclonal antibody COL-1 detection of CEACAMs in HeLa and Chang extracts. Lanes 1 and 4 contain 4 μg each of HeLa-CEA, -CEACAM1 and -CEACAM6. Lanes 2 and 5 contain 60 μg of total protein extract from unstimulated Chang cells, and lanes 3 and 6 contain 60 μg protein from stimulated Chang cells. Data show very low levels of CEACAM1 in unstimulated Chang cells, and only CEACAM1 is upregulated after IFN-γ treatment.

To confirm that IFN-γ treatment leads to CEACAM-surface expression on Chang cells and to assess the effect of other cytokines, flow cytometry analysis was carried out using several cytokines (Fig. 3A). IFN-γ increased CEACAM expression significantly on Chang cells within 24 h (Fig. 3A). During extended exposure to assess the time-course of cytokine stimulation, progressive increase in CEACAM expression was observed with IFN-γ over the first 24 h, which remained elevated over a 72 h period. However, tumour necrosis factor (TNF)-α or interleukin (IL)-1β had no such effect (Fig. 3B). As pili mediate binding and appear to augment Nm interactions to Chang cells via Opa proteins (Fig. 1B), and as CD46 may be involved in pilus binding (Kallstrom *et al*., 1997), we monitored CD46 expression levels also. None of the cytokines affected CD46-surface expression (Fig. 3C). That TNF-α preparations used were functionally active was ascertained using human umbilical vein endothelial cells (Huvecs), which were stimulated with 10 ng ml^−1^ TNF-α. As expected from previous reports, this treatment increased ICAM expression by about four-fold in Huvecs (Doukas and Pober, 1990) (data not illustrated).

*De novo* synthesis of CC1 has been reported in a variety of cells in response to cytokine stimulation (Dansky-Ullmann *et al*., 1995; Chen *et al*., 1996; Muenzner *et al*., 2000; Fahlgren *et al*., 2003). To assess whether *de novo* synthesis of CEACAM1 is induced in Chang cells by cytokine treatment, semiquantitative reverse transcription polymerase chain reaction (RT-PCR) was carried out. IFN-γ stimulation resulted in a five-fold increase in CEACAM-specific mRNA compared with unstimulated cells. TNF-α also increased the CEACAM1 transcript, but to a lesser extent (Fig. 3D and E). CEACAM1 expression has also been shown to be upregulated by *N. gonorrhoeae* binding to target cells (Muenzner *et al*., 2002). Therefore, we used adhesive and non-adhesive Nm phenotypes to study their effect on *ceacam* gene transcription in unstimulated Chang cells after a 3 h exposure to bacteria. The data show approximately two-fold increase in *ceacam1* transcript at 3 h, which was induced by Opa-expressing C751OpaD but not C751Op^–^ derivative (Fig. 3E).

The levels of distinct CEACAM members expressed in Chang cells prior to and after IFN-γ stimulation was determined by Western blotting using anti-CEACAM antibodies. Prior to the IFN-γ treatment, CEACAM expression was too low to be detected by Western blotting but this expression increased substantially following IFN-γ treatment and CEACAM1 was the only member of the family that was upregulated (Fig. 3F). No other proteins were detected by Western blotting using the polyclonal anti-CEACAM antibody AO115.

### Receptor modulation in human respiratory epithelial cell lines in response to distinct cytokines

In further experiments, the effect of several cytokines (pro-inflammatory IFN-γ, TNF-α, IL-1β and anti-inflammatory IL-4) on CEACAM1 as well as CD46 expression was investigated using a number of respiratory epithelial cell lines. Chang cells were used alongside for comparison. Several cell lines were processed simultaneously by flow cytometry to obtain relative mean fluorescence intensity (MFI) values using anti-CEACAM antibody AO115. Taking MFI as an indicator of the relative level of receptor present, it could be concluded that the basal levels of both CEACAM and CD46 expression varied between cell lines but CEACAM levels were more variable (Tables 2 and 3). With the exception of Detroit cells, the highest labelling was found on A549 cells. Following stimulation for 24 h, CEACAM expression increased significantly on all cell lines tested in response to IFN-γ (Table 2), but CD46 expression was largely unaltered (Table 3). With the exception of H292 treated with TNF-α, no significant change in CEACAM expression was observed in TNF-α-, IL-1β- or IL-4-treated cells (Table 2). IL-1β was also previously found not to induce CEACAM expression in colonic epithelial cells (Fahlgren *et al*., 2003).

**Table 2:** CEACAM expression in human respiratory cell lines and in Chang conjunctiva cells in response to cytokines.

Cell line	Non-stimulated	IFN-γ (100 U ml^−1^)	TNF-α (10 ng ml^−1^)	IL-1β (10 U ml^−1^)	IL-4 (100 U ml^−1^)
Chang conjunctival (*n* = 5)	7.2 ± 0.24	**17.0** ± 1.59	7.1 ± 0.22	6.7 ± 0.47	6.3 ± 0.39
		***P* = 0.01**	*P* = 0.88	*P* = 0.34	*P* = 0.09
H292 lung mucoepidermoid (*n* = 5)	10.8 ± 0.48	**16.3** ± 1.25	**15.5** ± 0.74	12.9 ± 0.73	11.0 ± 0.51
		***P* = 0.03**	***P* = 0.003**	*P* = 0.60	*P* = 0.20
A549 lung pneumocyte (*n* = 2)	26.1 ± 6.10	**50.8** ± 6.35	29.0 ± 5.80	33.9 ± 2.40	26.3 ± 2.80
		***P* = 0.01**	*P* = 0.85	*P* = 0.28	*P* = 0.96
Detroit 562 pharyngeal (*n* = 3)	413.9 ± 98.10	**451.3** ± 94.37	357.5 ± 72.54	459.0 ± 81.49	325.4 ± 85.16
		***P* = 0.04**	*P* = 0.45	*P* = 0.19	*P* = 0.08
HEp-2 laryngeal (*n* = 4)	5.2 ± 0.52	**7.7** ± 0.68	4.9 ± 0.27	5.0 ± 0.33	5.1 ± 0.28
		***P* = 0.05**	*P* = 0.56	*P* = 0.77	*P* = 0.73

Human epithelial cells were labelled with anti-CEACAM AO115 and anti-rabbit PE before or after cytokine stimulation for 24 h. The numbers are means of the MFI values with standard errors and the probability as calculated by Student's *t*-test. Numbers in bold show significant increases of MFI values from basal levels.

**Table 3:** CD46 expression in human respiratory cell lines and in Chang conjunctiva cells in response to cytokines.

Cell line	Non-stimulated	IFN-γ (100 U ml^−1^)	TNF-α (10 ng ml^−1^)	IL-1β (10 U ml^−1^)	IL-4 (100 U ml^−1^)
Chang conjunctival (*n* = 5)	61.2 ± 5.29	66.6 ± 5.37	61.0 ± 6.85	64.3 ± 4.22	61.5 ± 5.93
		*P* = 0.23	*P* = 0.96	*P* = 0.58	*P* = 0.94
H292 lung mucoepidermoid (*n* = 3)	46.2 ± 1.97	**52.6** ± 3.45	60.2 ± 4.93	57.6 ± 6.05	**55.0** ± 1.59
		***P* = 0.05**	*P* = 0.08	*P* = 0.11	***P* = 0.04**
A549 lung pneumocyte (*n* = 2)	115.4 ± 10.85	99.4 ± 3.50	122.2 ± 1.75	100.2 ± 7.35	101.1 ± 10.00
		*P* = 0.47	*P* = 0.68	*P* = 0.56	*P* = 0.62
Detroit 562 pharyngeal (*n* = 3)	65.7 ± 11.47	67.3 ± 13.86	55.8 ± 7.89	60.2 ± 7.72	50.8 ± 10.45
		*P* = 0.56	*P* = 0.13	*P* = 0.30	*P* = 0.11
HEp-2 laryngeal (*n* = 2)	55.4 ± 1.70	55.6 ± 4.15	58.7 ± 4.70	57.5 ± 6.65	56.7 ± 7.75
		*P* = 0.98	*P* = 0.47	*P* = 0.85	*P* = 0.92

The cells were labelled with anti-CD46 antibody J4-48 and anti-mouse PE. Other details are as in Table 2.

Consistent with this, C751OpaD exhibited increased binding only to IFN-γ-treated Chang cells but not TNF-α-, IL-1β- or IL-4-treated cells. No increase in C751Op^–^ association was observed following any of the cytokine treatments (data not shown).

### Studies on human respiratory epithelial cell line A549

To investigate Nm interactions with a respiratory cell line and to verify the adhesion-potentiating effect of IFN-γ, we used A549 in further experiments described below. This cell line responded to IFN-γ treatment with higher CEACAM response compared with other respiratory cells tested (Table 2).

#### 

##### 

###### Identification of key A549 adhesive phenotypes

**Fig. 4:**
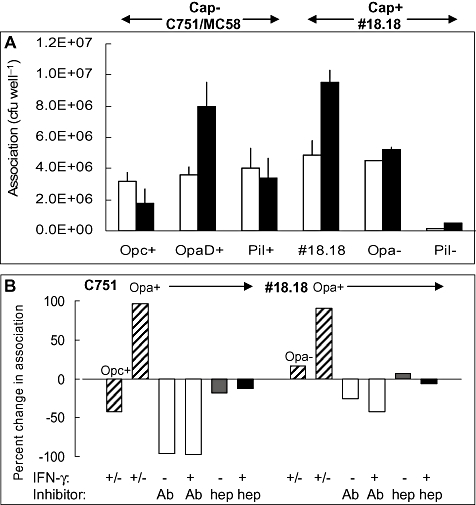
Adhesins and receptors involved in the interactions of various Nm phenotypes with unstimulated and IFN-γ-stimulated A549 cells. A549 monolayers were treated with IFN-γ (filled columns) or left untreated (blank columns), and adhesion of acapsulate or capsulate bacterial phenotypes was determined by viable count assays. Means and SEs from one typical experiment are shown (A).B. Per cent change in association of the acapsulate and capsulate variants to cells after IFN-γ treatment compared with untreated cells (shown as ‘+/−’) compiled from data of 3–4 independent experiments (hatched columns). In addition, data are also shown for the inhibition of interactions of Opa^+^ phenotypes in the absence or presence of anti-CEACAM antibody and heparin for both untreated and IFN-γ-treated cells (details as in legend to Fig. 2). SEs were within 25% of mean values shown.

We first examined the different phenotypes of Nm to assess which of the major adhesins played the most prominent role in A549 interactions. The data obtained were as observed for Chang conjunctiva epithelial cells, with Opa playing the most prominent role in both acapsulate and capsulate bacterial adhesion (cf. Figs 1 and 4). In addition, by the use of heparin and the CEACAM-blocking antibody AO115, it was apparent that CEACAMs were primarily involved in bacterial interactions with A549, with HSPG playing an insignificant role (Fig. 4B).

###### Identity of CEACAMs upregulated following IFN-γ treatment

**Fig. 5:**
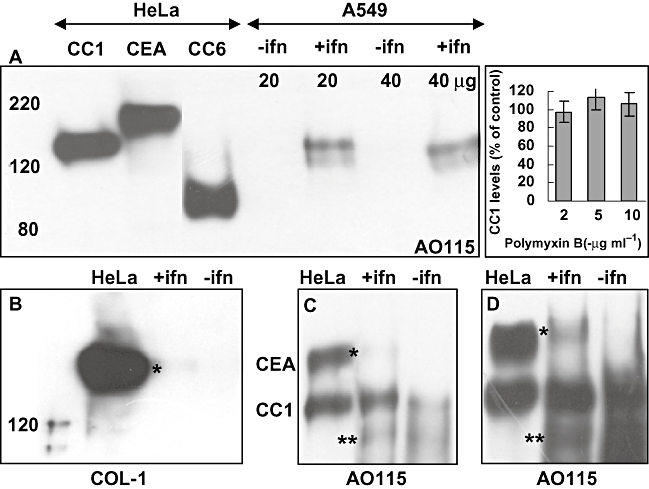
CEACAM protein expression in A549 before and after IFN-γ treatment. Extracts from transfected HeLa cells expressing CEACAM1 (CC1), CEA or CEACAM6 (CC6) were applied to individual wells as shown in A, and HeLa-CEA and -CEACAM1 mixtures were applied to single wells (lanes marked ‘HeLa’ in B–D) for comparison. A549 extracts (20–60 μg protein) were applied to individual wells as shown, and Western blots were developed using either AO115 to detect all CEACAMs expressed or COL-1 to detect CEA. Only CEACAM1 can be seen in A (20–40 μg loading) and only after IFN-γ treatment. On higher loading and further development, low levels of CEACAM1 were seen in unstimulated cells, and the protein band became more intense after the cytokine treatment (C). CEA was not detectable in unstimulated cells even on applying 60 μg of protein (D). After IFN-γ treatment, on higher loading a weak band can be seen with COL-1 (B). With 60 μg protein loading and much longer development, a band corresponding to CEA (*) can be seen more clearly (D). The bands shown by double asterisks are likely to be isoforms of CEACAM1. That CEACAM1 expression was not due to LPS contamination of IFN-γ preparations used, was demonstrated by incorporating increasing concentrations of polymyxin B (PB) to neutralize the effect of LPS (top right inset). Per cent CEACAM1 levels expressed were determined by densitometric analysis of CEACAM1 bands observed in the presence compared with the absence of PB.

CEACAM protein expression was monitored in A549 before and after IFN-γ treatment by electrophoresis and Western blot analyses. Untreated cells expressed lower levels of surface CEACAMs compared with IFN-γ-treated cells, as evidenced by immunofluorescence labelling of cells (data not illustrated), as well as by flow cytometry (Table 2). By Western blotting, no CEACAMs were observed on loading up to 40 μg protein of the unstimulated cell extract, whereas IFN-γ-stimulated cell extract (20 μg) produced a clear signal corresponding to CEACAM1 (Fig. 5A). On the other hand, at higher loading, a weak signal corresponding to CEACAM1 was seen in unstimulated cells (Fig. 5C), but CEA could only be seen in IFN-γ-treated cells (Fig. 5B–D).

###### Cellular invasion of IFN-γ-treated epithelial cells

**Fig. 6:**
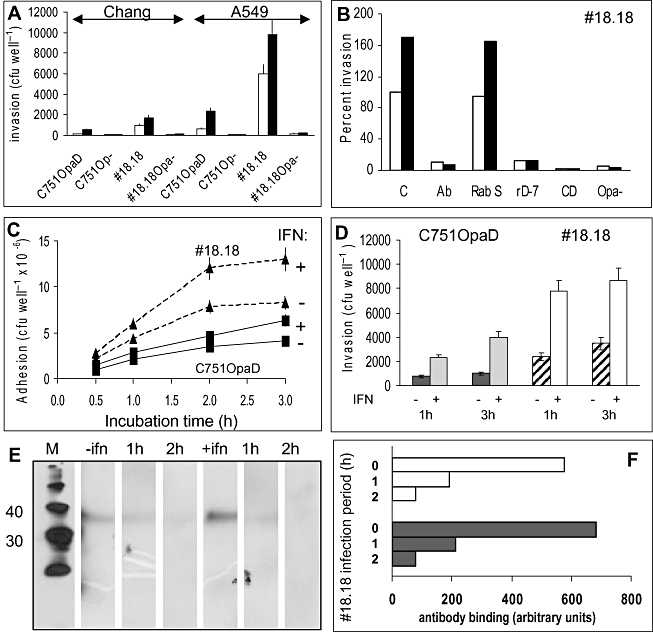
Invasion of IFN-γ-treated and untreated cells by Opa-deficient and proficient meningococcal phenotypes.A. Comparison of acapsulate and capsulate bacterial ability to invade Chang and A549 cells pretreated with IFN-γ (filled columns) or left untreated (blank columns).B. Inhibition of cellular invasion by #18.18 in the presence of anti-CEACAM antibody A0115 (Ab), CEACAM binding recombinant peptide (rD-7) or cytochalasin D (CD). CD treatment was used to demonstrate that on inhibition of cytoskeletal function, which usually inhibits uptake, no bacteria survived gentamicin treatment. Thus, any survivors of gentamicin treatment reported here represent internalized bacteria. Invasion levels of #18.18Opa^–^ are shown for comparison.C. Effect of IFN-γ treatment on the rate of cellular adhesion and invasion by acapsulate (squares) and capsulate (triangles) bacteria.D. Invasion of IFN-γ-treated or untreated cells by Nm at 1 and 3 h after infection. Means and SEs of one representative of several experiments are shown. To investigate NFκB activation by bacteria, IκB levels in the cytoplasmic fraction of cytokine-treated (+IFN) and untreated (–IFN) A549 cells were investigated by Western blot analysis shown in E. Lanes marked –IFN and +IFN contained extracts from cells which were not exposed to bacteria. Other lanes contained extracts from cells infected with #18.18 for 1 or 2 h as shown.F. Densitometric analysis of the bands observed in E (unstimulated cells: blank columns, stimulated cells: filled columns). Data are from one of two similar experiments.

When using Chang cells, we had observed only low levels of cellular invasion. However, with A549 cells, cellular invasion was significantly higher and increased further after IFN-γ treatment (Fig. 6A), a process that was completely abrogated by cytochalasin-D treatment (Fig. 6B). The essential role of CEACAMs in this process was demonstrated by the use of receptor-blocking agents (Fig. 6B).

###### Primary role of Opa proteins in cellular invasion and Opa–pili synergism

To consider the various phenotypic characteristics of capsulate meningococci and their influence on cellular interactions, the following observations may be emphasized: (i) #18.18 Pil^–^ (Cap^+^ Pil^–^ Opa^+^) bacteria did not adhere (Fig. 4A) or invade in significant numbers (not shown); and (ii) #18.18Opa^–^ (Cap^+^ Pil^+^ Opa^–^) bacteria adhered almost as well as #18.18 (Cap^+^, Pil^+^, Opa^+^) to unstimulated cells (Fig. 4A), but unlike #18.18, which invaded in significant numbers, no invasion of #18.18Opa^–^ was apparent (Fig. 6A and B). As cellular invasion was not observed with pil^–^ or Opa^–^ phenotypes of #18.18, it is clear that the expression of pili alone or Opa alone is insufficient to increase cellular invasion of capsulate bacteria.

###### Kinetics of cellular adhesion and invasion in resting and cytokine-stimulated A549 cells

In time-course adhesion experiments, bacterial binding was higher for IFN-γ-treated cells at early time points after infection, and cellular association appeared to reach a plateau after 2 h in the case of piliated bacteria (#18.18) (Fig. 6C). Cellular invasion was also accelerated and enhanced by IFN-γ treatment (Fig. 6D), particularly notable with #18.18. In the absence of the cytokine treatment, its invasion at 1 h was ≤ 70% that seen at 3 h. In cytokine-treated cells, invasion at 1 h was ≥ 90% of the enhanced invasion observed at 3 h, thus demonstrating the potential for accelerated bacterial infiltration of inflamed tissue. Moreover, for both capsulate and acapsulate bacteria, invasion increased by up to three-fold after 1 h in IFN-γ-treated compared with untreated cells.

### The role of NFκΒ in cellular adhesion and invasion in resting and cytokine-stimulated cells

Nuclear factor-kappa B is an important mediator of cellular response to bacteria (Naumann, 2000; Tato and Hunter, 2002) and may be an intermediate in some of the IFN-γ-induced signalling pathways (Zamanian-Daryoush *et al*., 2000). The activation of NFκB is induced on IκB release from its complex with NFκB; IκB is subsequently degraded. Therefore, to assess the role of this nuclear factor in the system under study, we examined IκB degradation in cytoplasmic fractions of both untreated and IFN-γ-treated A549 that were infected with #18.18. IκB was observed in uninfected cultures whether treated or untreated with the cytokine. However, within 1 h of bacterial infection, the levels of IκB decreased dramatically. Thus, bacterial infection induced NFκB activation in both unstimulated and cytokine-treated cells (Fig. 6E and F).

**Fig. 7:**
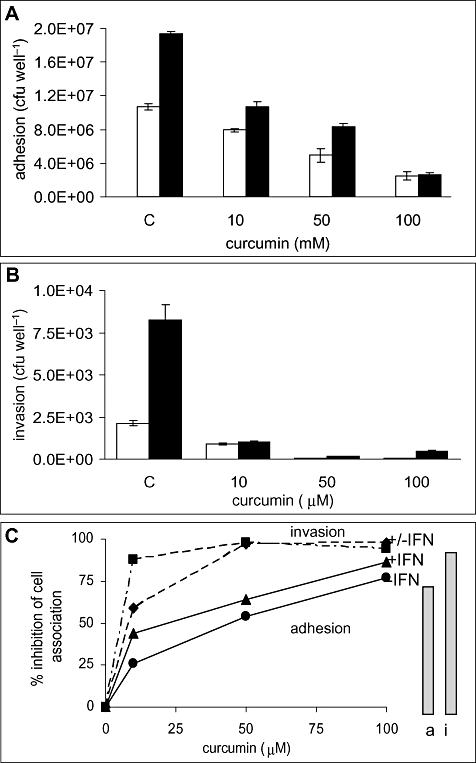
Curcumin-mediated inhibition of Opa-CEACAM-dependent adhesion and invasion of unstimulated and IFN-γ-stimulated target cells. A549 monolayers either exposed to IFN-γ (filled columns) or left untreated (blank columns) were pretreated with curcumin at increasing concentrations for 30 min prior to bacterial infection. The monolayers were washed carefully three times, and bacteria were added in Medium 199 supplemented only with 2% FCS. Cellular adhesion (A) and invasion (B) by #18.18 were assessed by viable counting. Means and SEs of triplicate estimations from one of several experiments are shown. Per cent inhibition of cell association and invasion at different curcumin concentrations in the case of #18.18 is compared in C. Inhibition of the cell association (a) and invasion (i) by acapsulate C751OpaD after 100 μM curcumin pretreatment of IFN-γ-treated cells are shown as bars in C.

In order to assess the role of this nuclear factor in meningococcal cellular adhesion and invasion of resting and IFN-γ-treated epithelial cells, we initially used diferuloylmethane or curcumin. The phytochemical has been shown to reduce activation of NFκΒ induced by *N. gonorrhoeae* and TNF-α (Wessler *et al*., 2005). Our experiments that had curcumin present at 50 μM throughout the meningococcal infection period, demonstrated a potent inhibition of Nm adhesion and invasion of A549. In these experiments, the possible effect of curcumin on bacterial expression of adhesins was monitored. Nm incubation with curcumin for 3 h had no effect on the levels of Opa, Opc or pilin expression as assessed by quantitative immunoblotting using specific monoclonal antibodies (data not shown). In further experiments, curcumin was used at a range of concentrations to pretreat resting or IFN-γ-stimulated A549 cells, which were then washed prior to infection with Nm. The compound reduced #18.18 cell association in a dose-dependent manner with unstimulated as well as stimulated A549 monolayers (Fig. 7A and C), and treatments with 10 μM curcumin virtually abrogated invasion (Fig. 7B and C). It is noteworthy that curcumin exerts is potent effect on cells during a 30 min preincubation period. Taken together, the effect of curcumin appears to be primarily via the effect on target cell monolayers, whether resting or pretreated with IFN-γ.

**Fig. 8:**
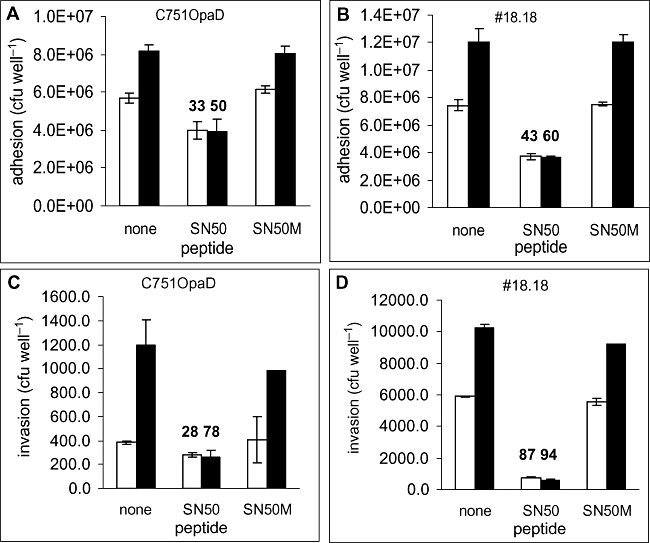
NFκB-SN50 peptide reduces cellular association and inhibits cellular invasion in unstimulated and IFN-γ-stimulated target cells. IFN-γ-treated cells (filled columns) or untreated cells (blank columns) were preincubated with NFκΒ nuclear translocation inhibitory peptide SN50 or a control peptide SN50M for 30 min prior to bacterial infection. Adhesion (A and B) and invasion (C and D) were assessed by viable counting. Mean values and SEs of triplicate estimations of representative experiments are shown. Numbers above SN50 bars are per cent inhibition of total cell association or invasion compared with controls without the peptide.

In order to address the role of NFκΒ more specifically, in further experiments, another NFκΒ antagonist was used. SN50 peptide contains the nuclear localization sequence (NLS residues 360–369) of the transcription factor NFκΒ p50 and prevents the nuclear translocation of NFκΒ specifically when used at low concentrations (Das *et al*., 2001). A control peptide (SN50M) which contains an inactive, mutated form of the nuclear localization sequence was used as a control. When the inhibitor was added to the cytokine-treated cells prior to bacterial infection,specific effects of SN50 but not the control peptide SN50M were observed (Fig. 8). With resting as well as cytokine-stimulated cells, inhibition of NFκΒ translocation to the nucleus with SN50 had a marked effect on cellular invasion (Fig. 8C and D). The effect on invasion was generally more pronounced than that on adhesion (Fig. 8A and B). The data are consistent with the notion that observed cellular adhesion is a product of pre-existing receptors as well as those that might be induced by bacterial adhesion. However, generally, cellular invasion requires the action of the NFκB induced after bacterial interactions with the target cells.

**Fig. 9:**
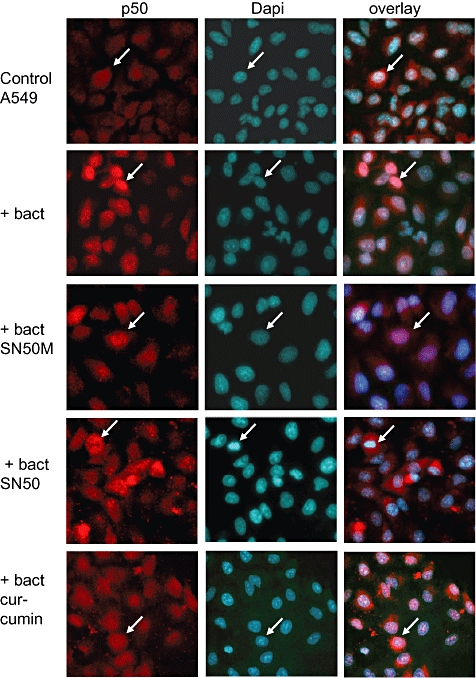
Immunofluorescence analysis of the localization of the p50 subunit of NFkB. A549 cells treated with NFkB inhibitors were infected with #18.18 (+bact). Monolayers were methanol fixed and labelled with anti-p50 antibody to localize the subunit of NFκB, or Dapi to localize the nuclei. The location of p50 in uninfected cells (Control) was largely cytoplasmic, whereas greater nuclear localization of p50 occurred in bacterially infected cells. This was not affected by SN50M control peptide, but was inhibited by SN50 or curcumin, resulting in cytoplasmic labelling of p50 in the latter two cases.

For a direct demonstration of the inhibition of NFκB localization into the nucleus in the presence of the inhibitors, immunofluorescence studies were undertaken using infected and uninfected A549 cultures, and the p50 subunit of NFκB was specifically labelled (Fig. 9). The results indicate that bacterial infection increases translocation of p50 into the nucleus and this is inhibited by curcumin and SN50 peptide, but not by SN50M (Fig. 9).

## Discussion

Epidemiological studies have highlighted the association of influenza and RSV outbreaks with increased incidence, as well as severity of certain respiratory bacterial infections. In the current studies, we have assessed whether inflammatory cytokines, particularly IFN-γ that may be augmented during viral infections, could render epithelia more vulnerable to meningococcal invasion. As epithelial cell lines vary with respect to their receptor expression and responses to bacterial challenge as well as to cytokines, we examined several cell lines of respiratory and ocular origin. Chang conjunctiva cells have been used previously *in vitro* studies of pathogenic Neisseriae and are known to express the receptors for the major adhesins, Opa, Opc and pili, of meningococci (Virji *et al*., 1993a,b;). Accordingly, we examined this cell line in the initial studies with IFN-γ-treated cells, which highlighted the role of Opa proteins in increased cellular interactions by Nm. Treatment of several target cell types with IFN-γ and other cytokines was also carried out to assess the effect on Nm cell association. In general, increased adhesion occurred only to cells treated with IFN-γ, with a small increase with some TNF-α-treated cells but never with cells treated with IL1-β or IL-4. Lack of availability of cytokine receptors on the apical surfaces of cell lines could be responsible for the lack of response to some cytokines, but the cell lines cultured on non-porous surfaces do not attain full polarization, and many cells, such as Chang cells, are not contact-inhibited and do not polarize in culture under the conditions used. Further analysis of four cell lines of respiratory origin identified A549 as the most IFN-γ-responsive cell line, with respect to CEACAM expression, which appeared to be the primary or major receptor family involved in Opa–mediated interactions with IFN-γ-stimulated cells.

One notable observation of meningococci–influenza synergism is the temporal relationship between the two infections: the latter occurs approximately 2 weeks following influenza infections (Cartwright *et al*., 1991; Hubert *et al*., 1992). The mechanisms that determine such temporal response remain unclear. However, excessive cytokine production has been observed in cases where bacterial infections occur in the late stages of viral infections (Nguyen and Biron, 1999; Beadling and Slifka, 2004). Combinations and levels of circulating cytokines could affect target cell stimulation significantly. A study on responses of cultured epithelial cells to cytokines demonstrated that CEACAM expression was stimulated two- to six-fold after 4 h and was sustained for 96 h after stimulation with IFN-γ (Fahlgren *et al*., 2003). In addition, IL-6 and IFN-γ at concentrations that had little effect when used separately resulted in synergistic effects on cellular CEACAM expression when used simultaneously (Dansky-Ullmann *et al*., 1995). Therefore, at optimum concentrations and duration of cytokines, host cell receptors targeted by bacteria may reach threshold levels required for bacterial invasion. We examined a combination of several cytokines, such as TNF-α, IL1-β and IL-4, with IFN-γ, but no significant difference over that of IFN-γ was observed (data not shown). Thus, in the current studies, all investigations were confined to the use of IFN-γ alone.

Pertinent to the current studies is a previous investigation that examined the effect of influenza A virus infection on meningococcal dissemination using virally challenged mice (Alonso *et al*., 2003). The authors reported that intranasal challenge of these mice with Nm leads to bacteraemia particularly at the peak of IFN-γ production (*c.* day 7). However, the mechanisms of dissemination may differ in mice compared with the human host, and the roles of Nm adhesins cannot be addressed in mice as Nm Opa proteins as well, as pili are human-specific adhesins. An earlier study (Read *et al*., 1999) examined adhesion of meningococci to influenzae B virus-infected nasopharyngeal organ cultures *in vitro* but did not observe increased adhesion. However, as the authors indicated, their study did not address the qualitative changes that may have occurred to allow invasion by a small number of bacteria. Also, under the *in vitro* infection conditions, whether cytokine stimulation occurred was not tested (Read *et al*., 1999).

In other investigations on human cell lines, TNF-α was used to induce the receptor expression in human umbilical vein endothelial cells and demonstrated increased Opa-mediated interactions of *Escherichia coli* expressing Nm Opa proteins (Muenzner *et al*., 2000). The studies did not address the effect on adhesion or invasion by capsulate meningococci. In considering meningococcal interactions with target tissue, the role of bacterial capsule must be addressed, as capsule downmodulates the adhesion via outer membrane proteins but is required for survival in the blood. Accordingly, whereas respiratory isolates may be capsulate or acapsulate, disseminated isolates are invariably capsulate. Hence, increased risk of dissemination may arise under conditions that permit cellular invasion by capsulate bacteria. In previous investigations, we have addressed the potential of target cell receptor densities on increased cellular invasion by capsulate/serum-resistant meningococci (Bradley *et al*., 2005; Rowe *et al*., 2007). Using transfected cell lines with inducible CEACAM expression (Tet-on system), increased invasion of capsulate bacteria was demonstrated in cells cultured with high levels of a tetracycline analogue used to induce the *ceacam-1* gene. Moreover, a threshold level of the receptor was required for efficient invasion. The studies also demonstrated that capsulate bacterial invasion was Opa-dependent but was significantly synergized by pili (Rowe *et al*., 2007).

In the current study, the aim was to investigate the roles of major surface adhesins of meningococci commonly expressed during infection, and as such, we have analysed #18.18 and its derivatives extensively, as it represents the phenotype most commonly isolated from disease cases. It is well established that in capsulate Nm, pili initiate initial contact with host cells via specific receptors. Pilus retraction over an extended period (*c*. 9 h) and capsule downmodulation is then thought to allow tight secondary binding (Pujol *et al*., 1999; Deghmane *et al*., 2000; Merz and So, 2000). However, in our examination of earlier events, pili appear to synergize the Opa-mediated binding of capsulate bacteria. In the absence of Opa, no cellular invasion was apparent, although adhesion did occur when bacteria were piliated. These observations are in accordance with our previous studies on transfected cell lines discussed above. Comparison of untreated and IFN-γ-treated Chang and A549 cells indicates the necessity for certain receptor density for significant cellular invasion by Opa-expressing Nm. How pili synergize Opa-dependent invasion requires further investigation, but long-range binding ability of pili that extend beyond capsule might be regarded as one likely property contributing to invasion potentiation by pili. That the internalization in the setting of high receptor density does not necessarily require significant downmodulation of capsule in #18.18 was demonstrated in our previous studies, in which internalized bacteria were shown to be as serum resistant as the inoculum, which would not be expected if the internalized bacteria had lost or had substantially reduced the capsule expression. Microscopic examination also led to the same conclusion (Rowe *et al*., 2007).

The receptor(s) recognized by meningococcal pili on human epithelial cells studied remain unknown. Although CD46, which is present on all nucleated cells, has been implicated in pilus-mediated binding of Nm and *N. gonorrhoeae* (Kallstrom *et al*., 1997), its involvement in pilus binding is controversial. An inverse relationship between total surface-exposed epithelial CD46 and adherence by *N. gonorrhoeae* has been reported (Tobiason and Seifert, 2001). The authors have also found no correlation between CD46 isoform expression and pilus-mediated gonococcal adherence. Using a panel of respiratory tract epithelial cell lines, we have found no change in CD46 expression in response to a range of cytokines over 72 h exposure. However, CD46 may be involved in pilus interactions at a secondary level (Lee *et al*., 2002; Weyand *et al*., 2006), and its role in the observed effects cannot be ruled out, despite the lack of its demonstrable upregulation.

Interferon-gamma has been shown to upregulate several epithelial cell receptors, including ICAM-1, MHC molecules and CEACAMs (Wong and Dorovini-Zis, 1992; Takahashi *et al*., 1993; Majuri *et al*., 1994; Dansky-Ullmann *et al*., 1995; Fahlgren *et al*., 2003). Further, pertinent to the current studies, is the suggestion that certain members of the CEACAM family play an important role in the innate immune defence (Hammarstrom and Baranov, 2001). The receptors can bind to several bacterial species in the gut and may clear mucosal pathogens through vesiculation or blebbing of microvilli with associated receptors (Hammarstrom and Baranov, 2001; Fahlgren *et al*., 2003). It is possible that over a longer time period, some receptor shedding may occur, but the current experiments show the early invasion potentiating role of CEACAMs in inflamed tissues rather than their potential protective role.

Interferon-gamma is reported to activate *ceacam-1* gene directly via the action of interferon regulatory factor-1 (IRF-1), which binds to interferon-sensitive response element (ISRE) present in the *ceacam-1*promoter (Chen *et al*., 1996). In addition, it has been observed that responses of cell lines to IFN-γ may also depend on the intrinsic levels of the receptor expressed; thus, cell lines with high intrinsic CEACAM levels did not respond to the cytokine with a further increase (Fahlgren *et al*., 2003). In the respiratory epithelial cells we studied, this was not always the case. Studies in progress in our laboratory are examining the effects of cytokines on receptor modulation of primary nasal epithelial cells. Preliminary studies show upregulation of a number of receptors, including CEACAMs (preliminary data presented at the International Pathogenic Neisseria Conference, Cairns, Australia, 2006).

In previous studies on *N. gonorrhoeae*, it was shown that bacterially induced NFκΒ may be involved in upregulation of CEACAM1 in epithelial cells in a TLR4-independent manner (Muenzner *et al*., 2002), but in endothelial cells, this involves TLR4 (Muenzner *et al*., 2001). IFN-γ may also induce its effect on target cells by activating NFκΒ via IFN-inducible protein kinase, RNA-dependent protein kinase (PKR). Activation of NFκΒ by PKR occurs in stress-induced signalling pathways (Zamanian-Daryoush *et al*., 2000). In the system under investigation, we observed that bacterial infection resulted in a significant activation of NFκB as assessed by degradation of IκB in the cytoplasm of infected cytokine-treated and untreated cells. To assess NFκB involvement in resting and IFN-γ-treated cells during bacterial adhesion and invasion, initially, curcumin was used as a potential inhibitor of NFκΒ. Curcumin, a phytochemical derived from turmeric (*Curcuma longa* Linn.), has been reported to decrease inflammatory response to bacteria and other effectors, and has been shown to reduce bacteria- (*N. gonorrhoeae, H. pylori*) and TNF-α-mediated activation of NFκΒ when used at concentrations between 20 and 100 μM (Foryst-Ludwig *et al*., 2004; Wessler *et al*., 2005). Curcumin appears to induce its antitumour activity also via its primary effect on NFκΒ (Thangapazham *et al*., 2006). In the current studies, the potent effect of curcumin was apparent in experiments using low concentrations (10 μM) to pretreat target cell. Even under these conditions, the agent reduced bacterial binding and nearly abrogated invasion. To further assess whether the effects of curcumin could be reproduced with other NFκB antagonists, NFκB-related peptide SN50 was used at 50 μg ml^−1^. At low concentrations, it inhibits nuclear translocation of NFκΒ specifically (Das *et al*., 2001). The inhibitory effects of the reagents on nuclear localization of the p50 subunit of NFκΒ were clearly observed by immunofluorescence analyses. Taken together, it can be deduced from the studies that NFκΒ indeed may control the cellular invasion of IFN-γ-treated as well as untreated cells by meningococci.

In conclusion, this study provides evidence that inflammatory stimulation of the respiratory tract epithelium may facilitate increased acapsulate and capsulate meningococcal adherence and invasion. This process appears to be CEACAM receptor density dependent and requires the expression of pili, but is likely to be independent of CD46 receptor density. Further, cellular invasion by capsulate Nm is totally dependent on Opa expression and can be downmodulated by CEACAM antagonists, such as the recombinant molecule rD-7, in addition to the inhibitors of NFκΒ. As CEACAM1 is the major CEA family member upregulated by IFN-γ and the receptor is targeted by other respiratory bacteria, including *H. influenzae* and *M. catarrhalis* (Virji *et al*., 2000; Hill and Virji, 2003), increased susceptibility to all these opportunistic bacteria following viral infection may be due to the upregulation of CEACAM1; a hypothesis that is under investigation. Overall, our data indicate that a combination of bacterial factors and human cellular phenotype may act in concert in some situations to increase host susceptibility to infection by frequent colonizers of the human respiratory tract*.*

## Experimental procedures

### Bacteria and growth conditions

Meningococcal culture conditions and enumeration have been described previously (Virji and Everson, 1981; Virji *et al*., 1991). The Nm serogroup A strain C751, and serogroup B strain MC58 and its mutants and phenotypic isolates, have been reported (Virji *et al*., 1993a; 1995b; McNeil and Virji, 1997; Bradley *et al*., 2005). All variants were phenotypically characterized for the expression of outer membrane components. Variants of strain C751 were non-piliated, and did not express capsule, and their lipopolysaccharide (LPS) is not intrinsically sialylated (Virji *et al*., 1993a). One isolate of strain MC58 (¢17) was a mutant with insertional mutation of the SiaD gene. It did not express Opa or Opc; its LPS was of L8 immunotype that cannot be sialylated but was piliated as assessed by negative stain electron microscopy (McNeil and Virji, 1997). MC58#18.18 and its derivatives were fully capsulate, with L3 LPS immunotype and expressed Opc (Virji *et al*., 1995b; McNeil and Virji, 1997; Bradley *et al*., 2005). The nomenclature and the relevant characteristics of the derivatives used in the current studies have been summarized in Table 1.

### Cell lines

Chang conjunctival and HEp-2 laryngeal cells were grown in Medium 199 supplemented with 10% fetal calf serum (FCS), 4 mM glutamine and 200 U ml^−1^ penicillin-streptomycin. H292 mucoepidermoid cells were grown in RPMI-1640 medium supplemented with 10% FCS, 4 mM glutamine, 1.5 g l^−1^ sodium bicarbonate, 5 g l^−1^ glucose, 1 mM sodium pyruvate, 10 mM Hepes buffer and 200 U ml^−1^ penicillin-streptomycin. A549 lung pneumocyte cells were grown in F-12 HAM nutrient medium supplemented with 10% FCS, 4 mM glutamine and 200 U ml^−1^ penicillin-streptomycin. Detroit 562 pharyngeal cells were grown in Minimum essential Eagle's medium supplemented with 4 mM glutamine, 1 mM sodium pyruvate and 200 U ml^−1^ penicillin-streptomycin.

### Antibodies and inhibitors

The polyclonal rabbit antiserum AO115 raised against human CEA was obtained from Dako (code no. AO115). AO115 reacts with other CEACAM subgroups, including CEACAM1 and CEACAM6. The mouse monoclonal antibody J4-48 reacts with CD46 (Serotec). COL-1 monoclonal antibody reacting with CEACAM3 and CEA but not other CEACAMs, was purchased from Zymed/Invitrogen. Appropriate secondary antibodies conjugated with phycoerythrin (PE) or horseradish peroxidise (HRP) were obtained from Sigma or Jackson laboratories. The recombinant CEACAM binding polypeptide (rD-7) based on the *M. catarrhalis* UspA1 CEACAM binding region, has been described previously (Hill *et al*., 2005). SN50 and SN50M were from Calbiochem/Merck, and curcumin (C1386) was from Sigma. In blocking experiments, the antibodies were used at 10–40 μg ml^−1^, and rD-7 was used at 1 μg ml^−1^. SN50 and SN50M were used at 50 μg ml^−1^ and curcumin at 10–100 μM concentrations. In all cases, the monolayer integrity and viability were monitored and were unaffected during the experimental time. None of the reagents affected bacterial viability or adhesin expression.

### Cytokine stimulation

Cytokines were added to confluent cells in Medium 199 (Sigma) supplemented with 2% (v/v) FCS (GibcoBRL) for 24 h except when indicated in the text. The concentrations used were based on those reported: IFN-γ (100 U ml^−1^) (Sigma); TNF-α 10 ng ml^−1^ (ICN); IL-1β 10 U ml^−1^ (Sigma); and IL-4 100 U ml^−1^ (Sigma). According to the manufacturers, LPS contamination of the cytokine preparations was < 1 pg ml^−1^.

### Measurement of bacterial association

Bacterial association and invasion was measured by viable count assays as previously described (Virji *et al*., 1991; Hill and Virji, 2003). Bacterial association after monolayer treatment with blocking antibodies, recombinant peptide or NFκB inhibitors was carried out using untreated or IFN-γ-treated confluent monolayers by preincubation with the blocking agents or inhibitors at concentrations indicated, usually for 30 min prior to infection of the monolayers with bacteria in Medium 199 supplemented with 2% FCS. Adhesion and invasion was then determined after a 3 h infection period. Multiplicity of infection (moi) of *c.* 200:1 was used throughout.

### Semiquantitative RT-PCR

RNA was extracted from whole-cell lysates using Purescript R-550 A (Gentra, Minnesota, USA), reverse transcribed using RETROScript first-strand synthesis kit (Ambion, Texas, USA), and relative levels of CEACAM1 transcript were measured by comparison with 18s rRNA levels. Ambion 18s primer : competimer pairs were used for semiquantification in a 1 : 12 ratio. The primers used were published previously (Wang *et al*., 1998).

### Measurement of surface receptor expression

Flow cytometry was used to measure the expression of CEACAM and CD46 on the cell lines as reported (Dixon *et al*., 1999). In brief, the cells were removed from culture wells, harvested by centrifugation, labelled with the appropriate primary and PE-conjugated secondary antibodies, and fixed with 1% (v/v) formal saline. A FACSCalibur (Becton Dickinson, Oxford, UK) was used for flow cytometry, and analysis was undertaken using WinMDI 2.8 (The Scripps Research Institute, La Jolla, California, USA) or Cellquest (Becton Dickinson).

### Western blotting

Cell lysates were prepared by lysis in 150 mM NaCl, 1% NP-40, 50 mM Tris, pH 8.0 containing protease inhibitor cocktail of 1 mM PMSF, 1 μM E-64, 100 μM pepstatin A and 60 nM bestatin. After 10 min lysis at 4°C, samples were transferred to eppendorf tubes and centrifuged to remove insoluble material. Supernatents were then used to estimate the concentration of proteins extracted by using BCA colorimetric method (Pierce). In total, 4–60 μg of proteins extracted was applied to 5% polyacrylamide gels and samples transferred to PVDF membranes (Millipore). Membranes were blocked in 10% milk in phosphate-buffered saline containing 0.05% Tween-20 (PBST) overnight at 4°C. All antibodies were diluted in 5% milk in PBST, and membranes were incubated for 1 h at room temperature, using predetermined optimum dilutions of antibodies. After incubation with secondary HRP-conjugated antibodies, the blots were developed using an advanced ECL system (Amersham) for chemiluminescent detection of CEACAMs.

### Demonstration of NFκB activation

A549 cells were infected with #18.18 (moi 200:1) for 1 h with or without the NFκB translocation inhibitory peptide SN50 or the control peptide SN50M present at 50 μg ml^−1^. Curcumin was used at 50 μM. At the end of the incubation, cells were methanol fixed and stained for the p50 subunit of NFκB using rabbit polyclonal antibody H-119 (Santa Cruz Biotechnology), followed by TRITC-conjugated secondary antibody. The nuclei were located using Dapi. For measurements of IkB levels, cytoplasmic fractions collected from cell lysates (as above) were subjected to SDS-PAGE and Western blotted using the antibody sc-371 (Santa Cruz).

### Statistics

All results were analysed using Microsoft Excel; two-tailed Student's *t*-test's were used throughout. A probability of 0.05 was taken to be significant.
